# Catastrophic Pediatric Stroke Following Attempted Transcatheter Ventricular Septal Defect Closure

**DOI:** 10.1016/j.jaccas.2026.108786

**Published:** 2026-06-17

**Authors:** Mohd Nur Aizat Mohd Yasim, Nishanan Panichnantho, Jirayut Jarutach, Supaporn Roymanee, Worakan Promphan

**Affiliations:** a Department of Pediatrics, Faculty of Medicine, Prince of Songkla University, Hat Yai, Songkhla, Thailand; b Pediatric Heart Center, Queen Sirikit National Institute of Child Health, College of Medicine, Rangsit University, Bangkok, Thailand

**Keywords:** cardiac catheterization, complications, pediatric stroke, transcatheter closure, ventricular septal defect

## Abstract

**Background:**

Periprocedural stroke is a rare but potentially catastrophic complication of pediatric cardiac catheterization.

**Case Summary:**

An 18-month-old girl with a perimembranous ventricular septal defect and progressive aortic valve prolapse underwent elective transcatheter closure. The procedure was abandoned after 2 unsuccessful device deployment attempts due to worsening aortic regurgitation and device instability. Two hours later, she developed acute neurological deterioration. Brain computed tomography demonstrated a large right hemispheric infarction combining mixed-density subdural collection, and uncal herniation. Emergency decompressive craniectomy with hematoma evacuation was performed. Evaluation for thrombophilia and coagulopathy was negative.

**Discussion:**

Thromboembolism related to subtherapeutic anticoagulation, contrast-related neurotoxicity, and patient vulnerability in the setting of procedural complexity were considered the most plausible mechanism.

**Take-Home Message:**

Strict anticoagulation monitoring, appropriate heparin dosing, careful contrast selection, and thoughtful patient selection are essential to improve the safety of pediatric transcatheter interventions.

## Visual Summary

**Figure:**
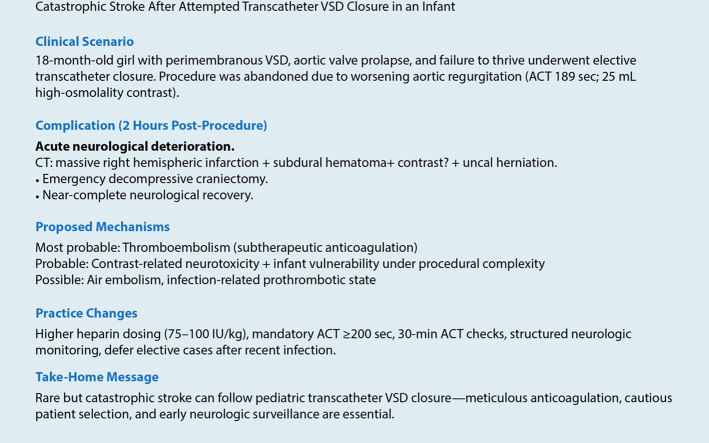

**Visual Summary:**
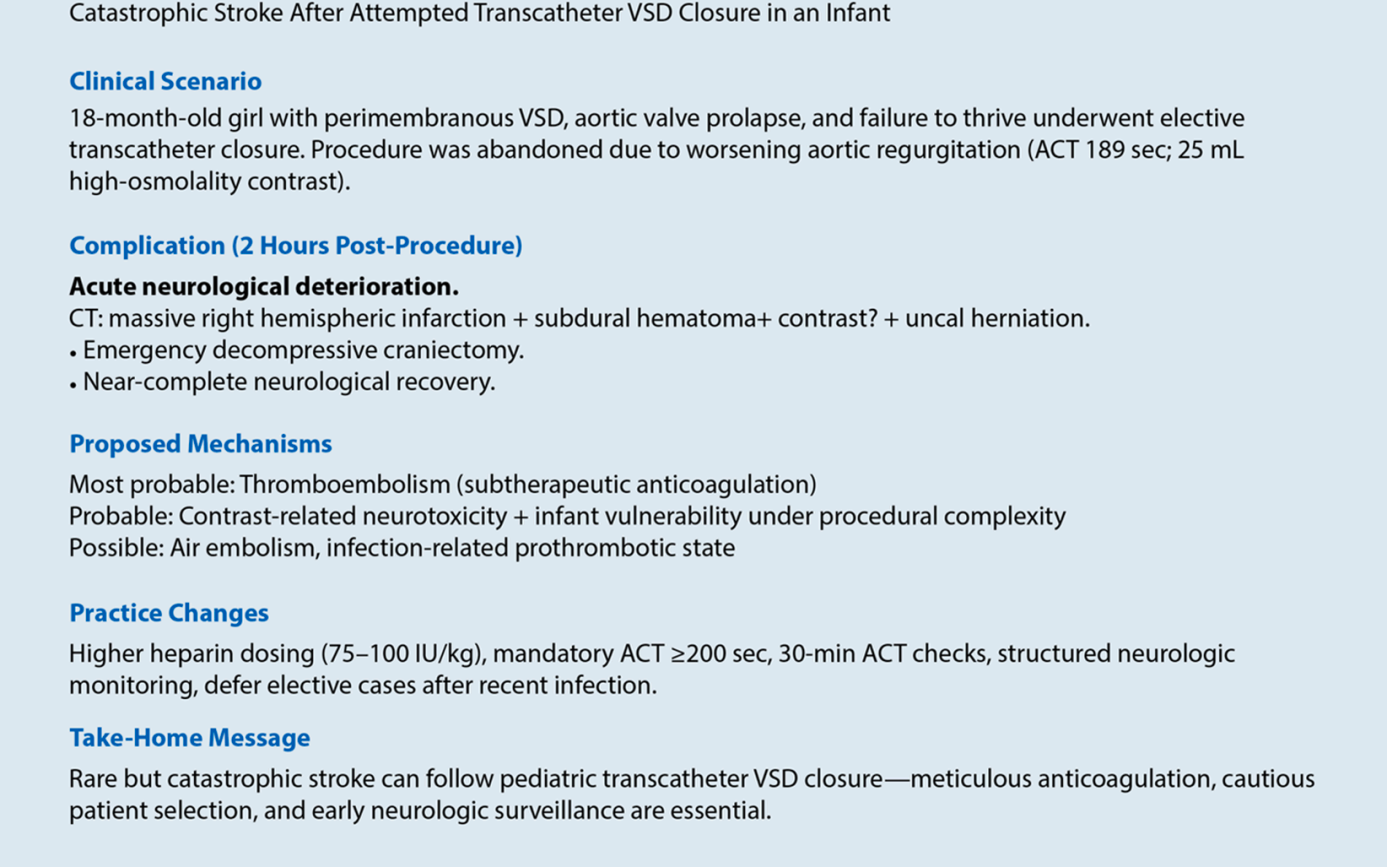
Summarizing the Essence of the Case

Transcatheter closure of ventricular septal defects (VSDs) is an established alternative to surgery in selected patients, particularly those with perimembranous or muscular defects. Although generally safe and effective, recognized complications include device embolization, residual shunts, valvular regurgitation, atrioventricular block, and hemolysis.^1^^,^^2^

**Take-Home Message**

• Strict anticoagulation monitoring, appropriate heparin dosing, careful contrast selection, and thoughtful patient selection are essential to improve the safety of pediatric transcatheter interventions.

Periprocedural stroke following pediatric cardiac catheterization is rare but potentially catastrophic, the incidence was 0.055% to 0.38%.^3^^,^^4^ Proposed mechanisms include thromboembolism, air embolism, paradoxical embolism, inadequate anticoagulation, device-related thrombosis, and hemodynamic instability. Infants are particularly vulnerable because of small vessel caliber, immature coagulation systems, and limited physiologic reserve.

We report a case of massive hemispheric ischemia and associated subdural hemorrhage shortly after attempted transcatheter VSD closure in an infant, highlighting potential mechanism and strategies to reduce procedural risk.

## Patient Presentation

An 18-month-old girl with a moderate-sized perimembranous VSD and secundum atrial septal defect was referred for intervention due to progressive aortic cusp prolapse with mild aortic regurgitation, left ventricular (LV) volume overload, and persistent failure to thrive.

Both surgical and transcatheter options, including potential complications, were discussed extensively by the congenital heart team. The family elected a transcatheter approach to avoid thoracotomy and cardiopulmonary bypass. Written informed consent for the procedure and publication was obtained. Institutional ethical approval was granted.

## Clinical Details

Two days before admission, the patient had mild rhinorrhea that resolved spontaneously. On admission, she was alert and hemodynamically stable (temperature 36.8 °C, heart rate 122 beats/min, respiration rate 32/min, blood pressure 87/58 mm Hg, and oxygen saturation 99% in room air). Weight and height were 7.8 kg and 74 cm, respectively. Cardiovascular examination revealed a grade 3/6 pansystolic murmur in the lower left sternal border. Chest radiography revealed cardiomegaly with pulmonary congestion. Transthoracic echocardiography demonstrated a perimembranous VSD partially restricted by a prolapsing aortic cusp (4.5 mm LV entry, 3 mm right ventricular [RV] exit), with associated mild aortic regurgitation. A 5 × 7-mm secundum atrial septal defect with adequate rims was also observed.

## Procedural Details

**Figure 1:**
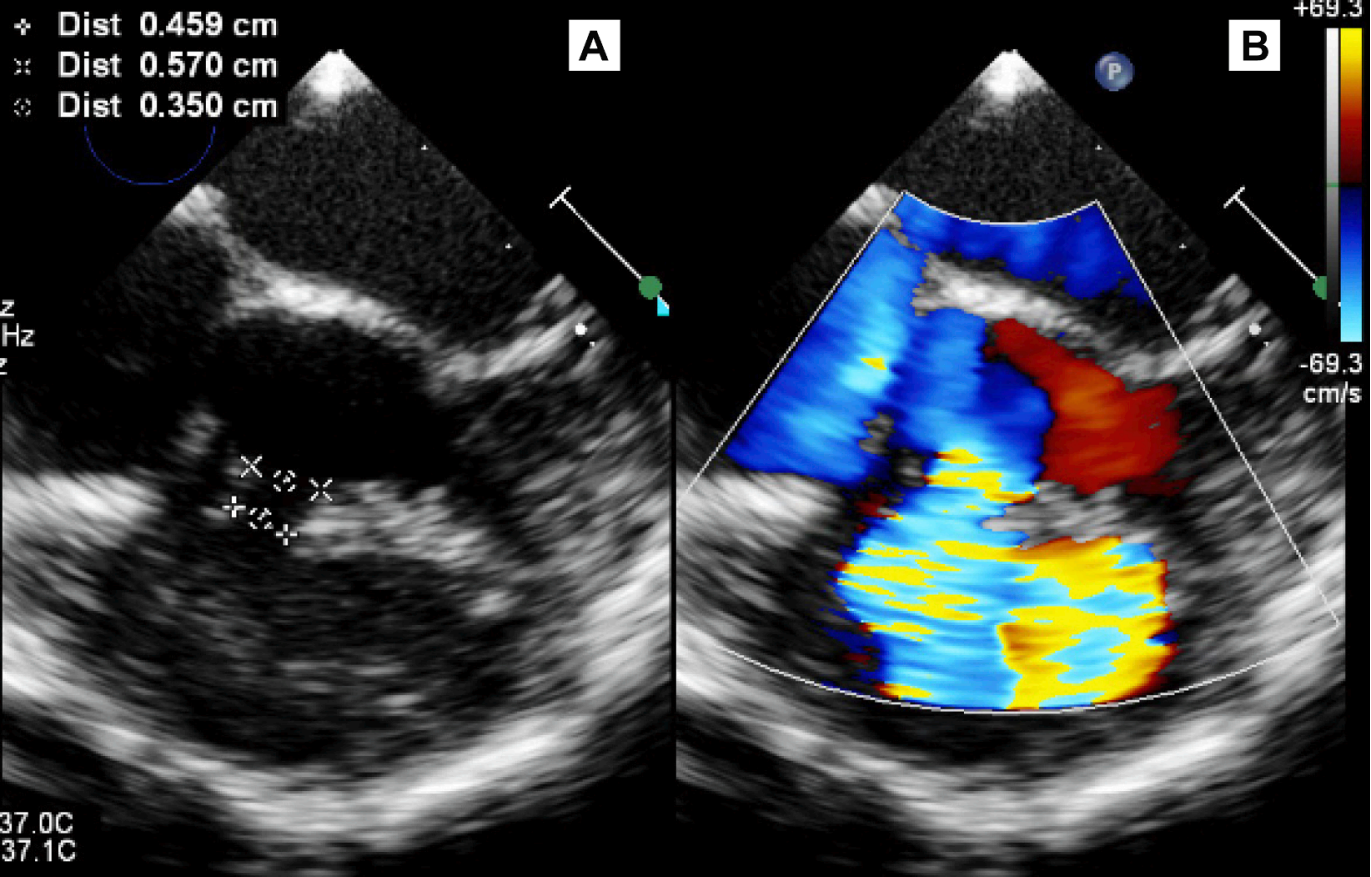
Transesophageal Echocardiography (Mid-Esophageal View, 0°) (A) Two-dimensional measurements showing defect size (left ventricular entry 5.7 mm, right ventricular exit 4.6 mm, length 3.5 mm). (B) Color Doppler demonstrating a left-to-right shunt.

The procedure was performed under general anesthesia with angiographic and transesophageal echocardiography (TEE) guidance. Preprocedural TEE confirmed a moderate perimembranous VSD (5.7 mm LV entry, 4.6 mm RV exit) (Figure 1). According to the institutional protocol, an initial heparin bolus of 50 IU/kg administered after arterial access, an additional bolus of 25 IU/kg was administered if the procedure exceeded 1 hour. Cefazolin 50 mg/kg was administered for antibiotic prophylaxis.

**Figure 2:**
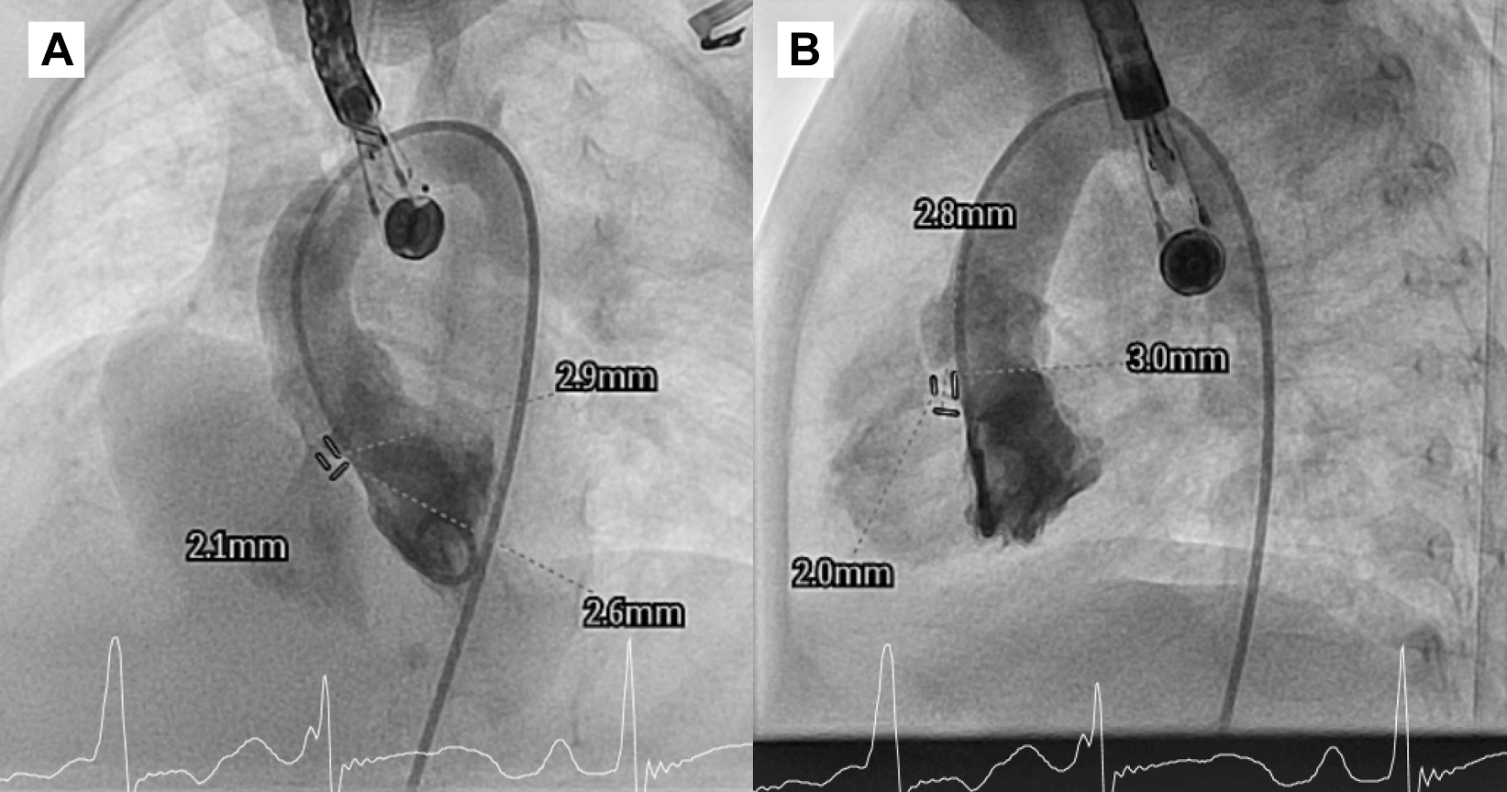
Left Ventricular Angiography (A) Left anterior oblique 33°, cranial 30°. (B) Lateral projection. A perimembranous VSD with measured dimensions and mild aortic valve prolapse is demonstrated.

Initial LV angiography at 1.5 mL/kg and aortography at 1 mL/kg with an injection pressure of 600 psi were successfully performed. Angiography revealed an RV exit diameter of 2.1 mm (Figure 2). No thrombus was visualized on intraprocedural TEE. Initially, a 4/4 mm Amplatzer Duct Occluder II (AGA Medical) was deployed retrogradely but was retrieved because of new-onset tricuspid and worsening aortic regurgitation. A second antegrade was attempted using the same device that worsened the aortic regurgitation and caused device instability, prompting procedural abandonment. Postprocedural TEE confirmed no new tricuspid regurgitation, the same degree of mild aortic valve prolapse and regurgitation, no intracardiac thrombus, and no pericardial effusion. The procedure time was 102 minutes, sheath-in and sheath-out time was 65 minutes, fluoroscopic time was 22 minutes, and total contrast usage was 25 mL of iopromide (Ultravist 370, Bayer Healthcare Pharmaceuticals). The patient was extubated after the procedure and was transferred in a stable condition.

## Clinical Course and Imaging

Two hours after the procedure, the patient developed progressive drowsiness, bradycardia, relative hypertension, and right pupil dilation, consistent with increased intracranial pressure (Glasgow Coma Score 10). She was reintubated and treated with hyperosmolar therapy with 3% sodium chloride, neuroprotective ventilation, and antiepileptic treatment initiated with phenobarbital, later transitioned to levetiracetam.

**Figure 3:**
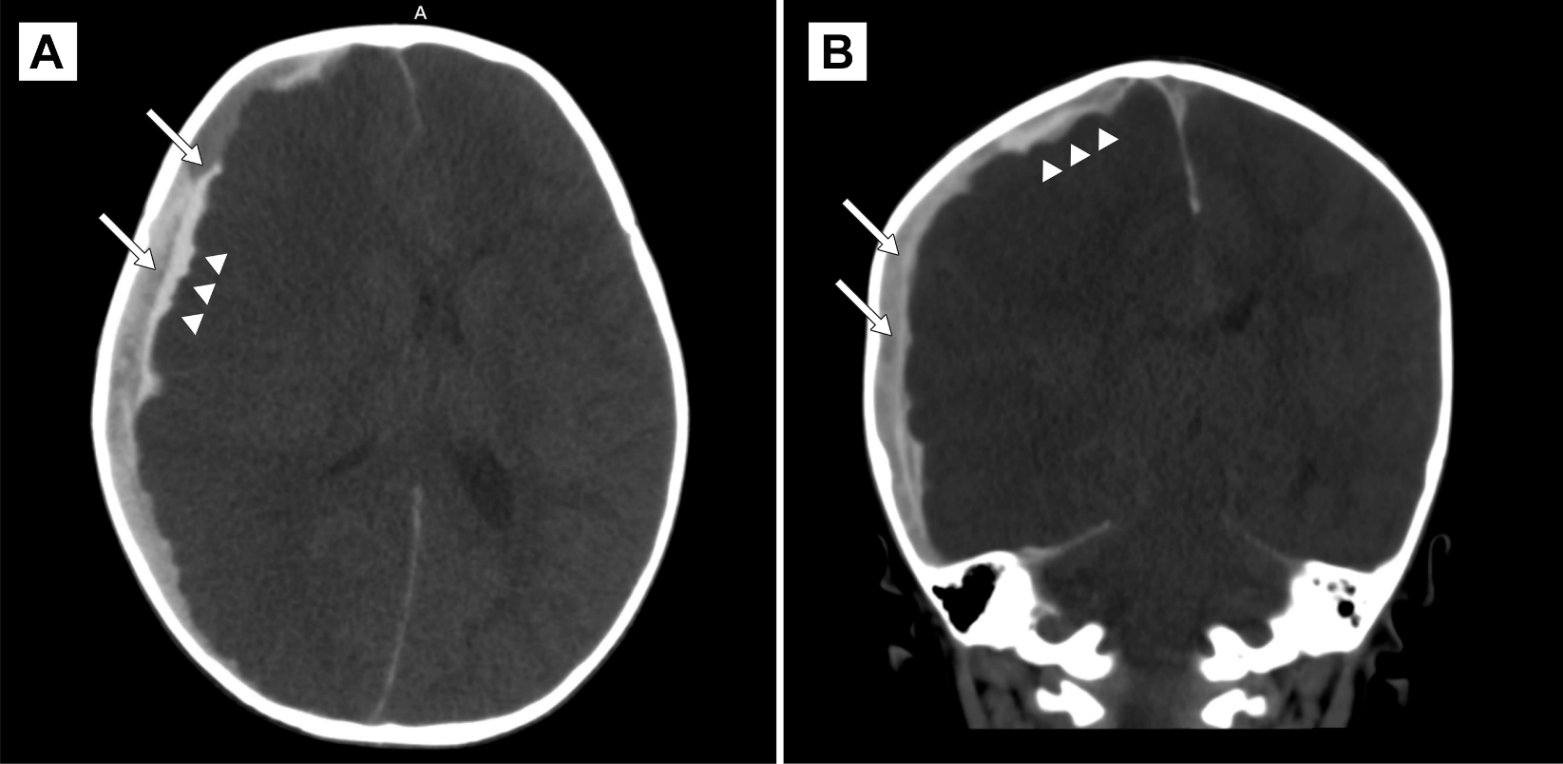
Noncontrast Computed Tomography of the Brain (3 Hours Post Procedure) (A) Axial plane. (B) Coronal plane. Large hypodense area in the right hemisphere consistent with acute infarction/ischemia, with associated mass effect. Two layers of mixed-density crescent-shaped collection along the right cerebral convexity likely represent contrast accumulation (arrowheads) and subdural hematoma/fluid (arrows).

Following the procedure, the differential diagnoses of acute neurological deterioration included air or thromboembolic infarction, cerebral hemorrhage, abnormal cerebral pathology, and contrast-induced encephalopathy. An emergent noncontrast brain computed tomography (CT) scan 3 hours after the procedure demonstrated a large area of hypodensity involving the edematous right cerebral hemisphere that could be acute infarction/ischemia, mixed-density collections along the right cerebral convexity, suggestive of contrast accumulation with possible subdural hematoma, and evidence of right uncal herniation (Figure 3).

Multidisciplinary consultations were sought with the pediatric neurology, hematology, and neurosurgery teams. CT findings revealed mixed-density lesions, raising the suspicion of both infarction and hemorrhage, precluding definitive identification of the exact mechanism of injury. Emergency right-sided decompressive craniectomy with evacuation of the subdural hematoma was performed. Intraoperatively, the dura was tense, and evacuation of a high-pressure mixed-density clot resulted in immediate decompression of the brain. No active source of bleeding was identified during the procedure. Follow-up neuroimaging confirmed infarct progression with no new hemorrhagic changes. Cerebral vasculature was normal. Comprehensive hematological evaluation, including thrombophilia and bleeding disorder panels, returned negative for inherited or familial coagulopathy. Following a 2-month hospital stay, the patient was discharged. At the follow-up, the patient demonstrated near-complete neurological recovery.

## Discussion

Although stroke after pediatric cardiac catheterization is rare, outcomes can be catastrophic. Prior studies identify balloon dilation for pulmonary vein stenosis and systemic–pulmonary collateral interventions as the most commonly associated procedures.^5^ In transcatheter VSD closure, both ischemic and hemorrhagic strokes have been described, typically related to paradoxical embolism, neurovascular abnormalities, hemodynamic instability, or procedural complexity.

In this case, we propose the following prioritized etiologic considerations.

### Thromboembolism (most probable)

Our institutional protocol uses an initial heparin bolus of 50 IU/kg, with activated clotting time (ACT) measurement at 1 hour and administration of an additional 25 IU/kg if the procedure is ongoing. In this case, with a vascular access time of 1 hour, only a single ACT measurement was obtained (189 seconds before sheath removal), which was below the recommended threshold of 200- to 250-second threshold.^6^^,^^7^ Subtherapeutic anticoagulation may have facilitated thrombus formation. This case prompted a change in institutional practice of a higher initial heparin dose (75-100 IU/kg), maintenance of ACT ≥200 seconds, and need for more frequent ACT monitoring (every 30 minutes), particularly in infants, who have a shorter heparin half-life.

### Contrast-related injury (probable contributor)

**Figure 4:**
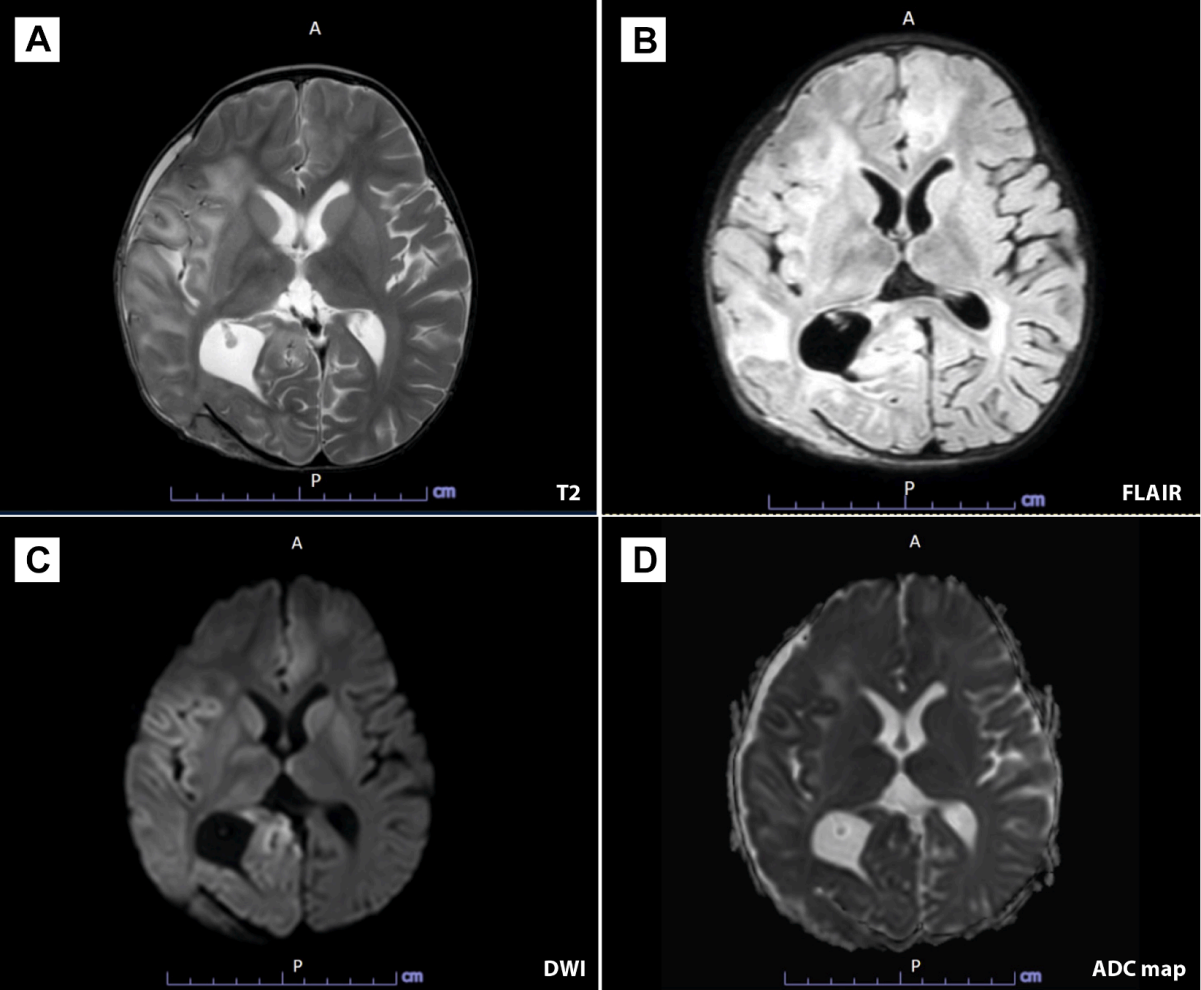
Magnetic Resonance Imaging Brain (Day 8) T2-weighted (T2) and fluid-attenuated inversion recovery (FLAIR) images (A and B) demonstrate hyperintense lesions involving the cortex and subcortical white matter of the right occipital, right parietotemporal, and left frontal lobes. Diffusion-weighted image (DWI) and apparent diffusion coefficient (ADC) map images (C and D) show cortical restricted diffusion consistent with acute infarction. The adjacent white matter demonstrates no diffusion restriction, suggestive of vasogenic edema.

The early CT findings demonstrated a large ischemic territory with mixed hemorrhagic features within 3 hours of symptom onset, an atypical pattern raising concern for contrast-related neurotoxicity. Follow-up magnetic resonance imaging on day 8 showed vasogenic edema (Figure 4), which may support this mechanism. Proposed mechanisms include blood-brain barrier disruption from hyperosmolar agents, direct neuronal toxicity, and transient vasoconstriction.^8^ Although iopromide (Ultravist 370; 769 mOsm/kg) is widely used, its relative high osmolality may pose greater risk in infants with immature brains and limited physiological reserve. Consequently, our institution has adopted lower-osmolality iohexol (Omnipaque 300; 684 mOsm/kg) for children younger than 2 years of age.

### Patient vulnerability (probable contributor)

Infants are inherently vulnerable due to small vessel caliber, immature cerebral autoregulation, and limited physiological reserve. Although VSD is generally considered a simple lesion, transcatheter VSD closure can be technically complex, and may involve hemodynamic compromise during arteriovenous looping and catheter manipulation across the tricuspid valve. In patients with weight <10 kg, or with associated aortic valve prolapse or regurgitation, surgical repair may offer a more predictable safety profile and should be strongly considered.

### Air embolism (possible)

Despite meticulous de-airing being routine practice, the small caliber of infant cerebral vessels increases susceptibility to even minimal air emboli. Strict adherence to air-elimination protocols remains essential.

### Infection-related vasculopathy (possible)

The recent upper respiratory infection may have contributed to a transient prothrombotic state through cytokine-mediated endothelial activation, as recent infections are associated with a modest but measurable increase in stroke risk.^9^ Although pediatric guidelines do not define optimal timing for elective procedures after viral illness, this case prompted a change in institutional practice to defer elective catheterization for 2 to 4 weeks in high-risk children.

### Study limitations

This single-case observation limits generalizability. The proposed mechanism was inferred from clinical and imaging correlations, as definitive differentiation was constrained by the absence of early diffusion magnetic resonance imaging and Hounsfield unit analysis. No intraprocedural thrombus was directly visualized.

## Conclusions

Although transcatheter VSD closure is generally safe, catastrophic stroke can occur. In this case, thromboembolism associated with subtherapeutic anticoagulation, contrast-related injury, and patient vulnerability during procedural complexity were the most plausible mechanisms. Strict ACT monitoring, optimized heparin dosing, careful patient selection, and continuous reassessment of procedural risk-benefit balance are essential to improve safety in pediatric transcatheter interventions.

## Funding Support and Author Disclosures

The authors have reported that they have no relationships relevant to the contents of this paper to disclose.
